# Dielectric Elastomers with Liquid Metal and Polydopamine-Coated Graphene Oxide Inclusions

**DOI:** 10.1021/acsami.2c21994

**Published:** 2023-05-15

**Authors:** Yafeng Hu, Carmel Majidi

**Affiliations:** † Department of Materials Science & Engineering, Carnegie Mellon University, Pittsburgh, Pennsylvania 15213, United States; ‡ Department of Mechanical Engineering, Carnegie Mellon University, Pittsburgh, Pennsylvania 15213, United States

**Keywords:** liquid metal, dielectric elastomer, graphene oxide, eutectic gallium−indium, high-*k* dielectric, reduced graphene oxide, polydimethylsiloxane, elastomer composite

## Abstract

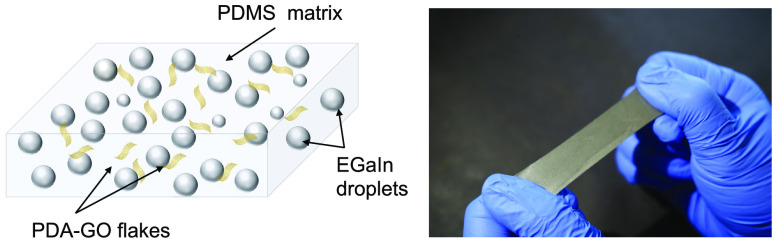

Suspending microscale droplets of liquid metals like eutectic gallium–indium (EGaIn) in polydimethylsiloxane (PDMS) has been shown to dramatically enhance electrical permittivity without sacrificing the elasticity of the host PDMS matrix. However, increasing the dielectric constant of EGaIn–PDMS composites beyond previously reported values requires high EGaIn loading fractions (>50% by volume) that can result in substantial increases in density and loss of material integrity. In this work, we enhance permittivity without further increasing EGaIn loading by incorporating polydopamine (PDA)-coated graphene oxide (GO) and partially reduced GO. In particular, we show that the combination of EGaIn and PDA-GO within a PDMS matrix results in an elastomer composite with a high dielectric constant (∼10–57), a low dissipation factor (∼0.01), and rubber-like compliance and elasticity.

## Introduction

Soft materials that are capable of sensing,^1−3^ actuation,^4−7^ and energy harvesting^8−10^ have an important role for emerging applications in wearable electronics^11−13^ and soft robotics.^14−16^ Among these soft transducer materials, dielectric elastomers have been especially popular due to their mechanical compliance, extreme elastic deformability, and compatibility with low-power electronics.^16−18^ Rubbery polymers like polydimethylsiloxane (PDMS),^19^ polyurethane,^20^ polyacrylates,^21^ and styrenic block copolymers (e.g., SEBS^22^ and SIS^12,23^) are widely used as dielectric elastomers because of their low elastic modulus and robust electrical insulating properties. However, compared to many classes of ceramics, fluids, and insulating crystalline materials, elastomers have a relatively low dielectric constant, which means that there is limited coupling between voltage and electrical current or charge. Embedding elastomers with nano- or microscale inclusions of high-dielectric constant ceramics such as BaTiO_3_,^24^ TiO_2_,^25^ and Al_2_O_3_^26^ is one common method for improving electrical permittivity. However, these composites suffer from degraded mechanical compliance, stretchability, and elastic resilience. Alternatively, conductive filler-like Ag flakes,^27^ CNT,^28^ or graphene oxide^29,30^ can be embedded within soft elastomers to create an “artificial dielectric”. Because the inclusions are electrically conductive, they effectively have an infinitely high electrical permittivity, leading to a dramatic amplification of the dielectric constant^31^ and showing the potential application in pressure sensing^32^ and human motion monitoring.^33^ However, because they are stiff compared to the matrix material, adding these conductive filler also leads to significant degradation of the elastic properties of the elastomer.

In recent years, there has been growing interest in the use of liquid metal (LM) alloys like eutectic gallium–indium (EGaIn) as a filler material for tailoring the electrical and thermal properties of soft materials.^5,34−38^ Unlike BaTiO_3_, Ag flakes, and other rigid filler materials,^39^ EGaIn is fluidic and can be incorporated in the form of microscale droplets that deform with the surrounding elastomer without introducing stress concentrations or significant mechanical resistance. Moreover, because these droplet inclusions are electrically conductive, they can be used to create artificial dielectrics with electrical permittivities that are 1 order of magnitude greater than that of an unfilled elastomer.^34,36^

However, further enhancement of permittivity requires high concentrations of EGaIn droplets in excess of 50 vol %. This results in significant increases in mass density compared to those of other dielectric composites that utilize filler materials with a lower specific gravity or that require smaller volume fractions. Moreover, for very high EGaIn volume fractions, the composite can lose its material integrity and rubber-like elasticity. A potential solution is to combine EGaIn droplets with a low concentration of additional filler material that has a lower density and/or a higher aspect ratio to enhance electrical permittivity without significantly altering mass density or elasticity.^40^

**Figure 1:**
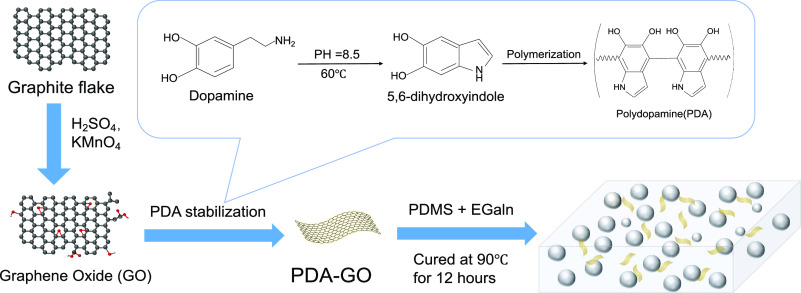
Schematic illustration of the preparation of PDA-GO and PDA-GO/LM/PDMS composites.

**Figure 2:**
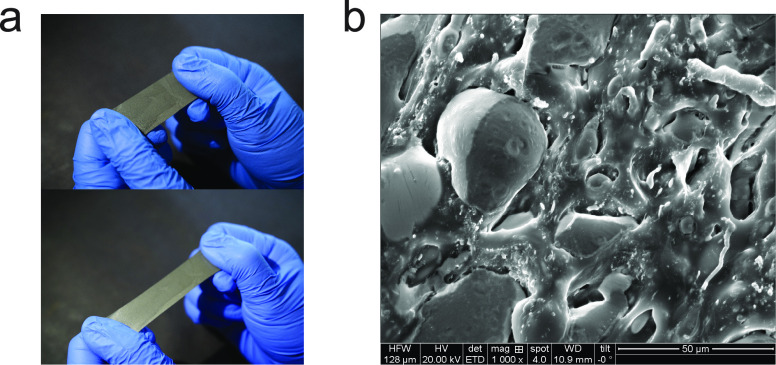
(a) Photographs showing the stretchability of PDA-GO/LM/PDMS composites. (b) Cross-sectional SEM image of PDA-GO/LM/PDMS composites.

In this study, we introduce a class of dielectric elastomer composites composed of polydimethylsiloxane (PDMS) rubber embedded with a suspension of EGaIn droplets as LM filler and polydopamine (PDA)-coated graphene oxide (GO) and partially reduced GO (rGO). The dielectric elastomer prepared by shear mixing is described in Figure 1, and the photographs and the cross-sectional SEM images of the PDA-GO/LM/PDMS composites are shown in Figure 2. Combining EGaIn with a relatively low concentration of PDA-GO allows for a further increase in electrical permittivity (∼10–57) while preserving mass density and maintaining a strain limit and elastic modulus similar to those of the unfilled PDMS. PDA is utilized because it can self-polymerize and adhere to GO and is capable of partially reducing GO during its self-polymerization process.^41^ Moreover, its aromatic groups and hydrogen bonds are highly polarizable, which helps improve dielectric constants and breakdown strengths.^42,43^ For example, Song et al. added PDA-coated BaTiO_3_ nanofibers into epoxy and confirmed the dielectric constant of the obtained composites reached ∼17 at 10 kHz.^42^ Yang et al. synthesized a novel BaTiO_3_–poly(dopamine)–silver nanoparticle that was used to improve the dielectric properties of nitrile–butadiene rubber (NBR) nanocomposites. The dielectric constant of the nanocomposite increases to 21.5 at 1 Hz.^44^ We investigate the dielectric constant and dissipation factor of PDA-GO/PDMS composites with and without microscale droplets of EGaIn to elucidate the contributions of the various filler components. We also examine the microstructure and mechanical properties of PDA-GO/PDMS and PDA-GO/LM/PDMS composites. Lastly, we demonstrate the practical use of these material systems as soft matter transducers by creating a parallel-plate capacitor that can couple changes in electrical field and mechanical deformation.

## Results and Discussion

### Characterization of Graphene Oxide (GO) and Polydopamine-Coated Graphene Oxide (PDA-GO)

Dopamine can simultaneously be used to coat the GO sheets and to also partially reduce GO during self-polymerization within a weak base solution.^41,45^ Fourier transform infrared (FTIR) spectroscopy is used to reveal the functional groups contained in PDA-GO. FTIR spectra of GO and PDA-GO are presented in Figure S1. The typical peaks of GO appear at 1732, 1624, 1213, and 1047 cm^–1^ corresponding to C=O stretching of COOH groups, the C=O stretching vibration, deformation of C–O, and C–O vibrations from alkoxy groups, respectively. After self-polymerization of dopamine, the intensity of the 1732 cm^–1^ peak becomes lower and the peak of 1624 cm^–1^ disappears, indicating that the concentration of the oxygen-containing group is partially reduced. Meanwhile, two new peaks at 1570 and 1361 cm^–1^ are observed that represent the stretching vibrations of N–H of amide and C–N vibration, respectively, proving the reaction between the epoxide group and the amine group and illustrating the formation of PDA-functionalized GO (PDA-GO) sheets.^46−48^Figure S2 displays the SEM images of PDA-GO and the elemental analysis. The elemental mapping results confirmed the presence of carbon (b), oxygen (c), and nitrogen (d) in PDA-GO.

### Dielectric Properties of Composites

**Figure 3:**
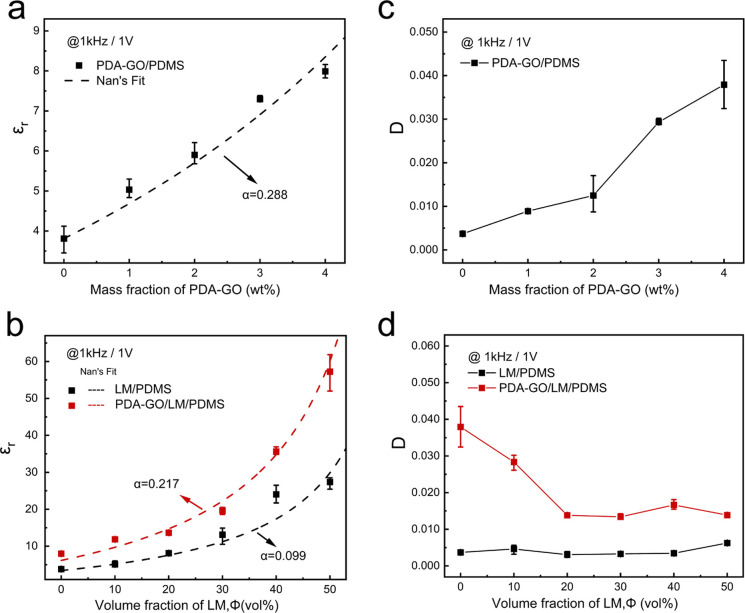
Evaluation of PDMS composites with different filler dielectric properties. Plots of (a) the dielectric constant and (c) the dissipation factor of the PDA-GO/PDMS composite with different PDA-GO contents at 1 kHz. The dashed lines are the theoretical fits with an effective medium theory. Plots of (b) the dielectric constant and (d) the dissipation factor of the LM/PDMS composite with and without PDA-GO added vs the volume fraction loading of liquid metal at 1 kHz. The dashed lines are the theoretical fits with an effective medium theory.

We first evaluated the dielectric constant (ε_r_) and dissipation factor (*D*) of PDA-GO/PDMS composites as a function of PDA-GO content at a frequency of 1 kHz and 0% strain to examine the dielectric properties and the ability to store charge (Figure 3a,c). The dielectric constant is found to increase with the PDA-GO mass fraction, reaching 7.99 at the highest concentration. This corresponds to an enhancement of 109.6% compared to the dielectric constant of pure PDMS. However, the dissipation factor also increases with the amount of PDA-GO, which is 10 times higher than that of pure PDMS at a PDA-GO concentration of 4 wt %. This enhancement of the dielectric constant can be explained by the formation of microcapacitors that are randomly distributed within the matrix.^49^ Increasing the PDA-GO filler content in PDMS results in a higher concentration of these microcapacitors, leading to enhanced electrical permittivity. The incremental increase in dissipation factor is attributed to the increase in the conductivity of the PDA-modified graphene oxide filler with an increase in the degree of reduction, which acts as a transmission path for electrons under the electric filed.^41^

Before the examination of the dielectric behavior of the PDA-GO/LM/PDMS composite, the permittivity and dielectric loss of the LM/PDMS composites are also tested for comparison, as shown in panels b and d of Figure 3 (black dots) at a frequency of 1 kHz. EGaIn is used as the LM filler. Unlike rigid fillers, LM-embedded elastomer composites combine low mechanical stiffness and high elastic stretchability with a high dielectric constant and a low dissipation factor.^34^ The permittivity increases with LM loading, and the dielectric constant increased from 3.81 to 27.37 with 50 vol % LM added. However, the dissipation factor of the LM/PDMS composites only slightly increases from 0.003 to 0.006, which is still low.

To further enhance the dielectric permittivity of the LM/PDMS composite, 4 wt % PDA-GO is added to the LM-embedded elastomer. Panels b and d of Figure 3 (red dots) show the dielectric properties of the PDA-GO/LM/PDMS composite versus the volume fraction of liquid metal. The plot shows that as the concentration of LM increases, the increase in dielectric constant is not linear. For the PDA-GO/LM/PDMS system, the dielectric constant of the composite with a Φ_LM_ of 50 vol % and a Φ_PDA-GO_ of 4 wt % is 57.23, which increases >1400% as compared to pristine PDMS. Meanwhile, with LM added, the distance between each PDA-GO sheet increases, preventing the formation of conductive pathways leading to a decrease in the dissipation factor (∼0.013 at the highest LM concentration). A similar phenomenon is also observed in a graphene/BaTiO_3_/P(VDF-HFP) composite where incorporating BaTiO_3_ platelets effectively suppresses the dielectric loss.^50^

To better understand the dielectric behavior of PDA-GO/LM/PDMS composites, we examine the influence of PDA-GO and LM filler concentration on the effective permittivity (ε_rc_) of the composite. Specifically, we fit experimental measurements of ε_rc_ with an effective medium theory (EMT) developed by Nan et al.:^51^
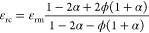
1where ε_rc_ and ε_rm_ are the dielectric constant of the composite and the matrix, respectively. α is a dimensionless value that is associated with droplet size and droplet shape. Smaller inclusions correspond to a larger α.^36,51^ Nan’s theory is based on multiscattering theory, which considers the properties of the matrix and reinforcement, particle amount, particle size, and size distribution to develop a general effective medium approximation that can be widely applicable.^51^ To make sure adding filler increases the relative permittivity of this system, α needs to be <0.5. For all of the composites, we use 3.81 as the dielectric constant of the matrix, which corresponds to the electrical permittivity of pristine PDMS. The filler particles are electrically conductive, so they are assigned an infinite value for their dielectric constant. The volume fraction of 4 wt % PDA-GO is added to the equation when fitting the PDA-GO/LM/PDMS composite curve. The fitting data match well with the experimental data for reasonable values of interfacial factor α, and this indicates that PDA-GO and LM co-filler can be considered as a single phase in the composite system.

Other potential EMT models, including the Maxwell–Garnett (MG), modified Maxwell–Garnett, and Bruggeman formulations (Figures S5 and S6), were applied to predict the theoretical dielectric constant of the composites.^52,53^ For the LM/PDMS composites, all of the EMT models fit reasonably well with the experimental data except for the Maxwell–Garnett model, which suggests that the shape of the LM and the local-field effects have an influence on the dielectric response.^54^ For the PDA-GO/PDMS composites, the Maxwell–Garnett and Bruggeman models give considerably lower values for the dielectric constant of composites. However, Nan’s formulation and the modified Maxwell–Garnett model consider morphology fitting factor α and *n* and are shown to fit the experimental data better than the other two models. In general, we expect that the morphology of PDA-GO inclusions will have a significant influence on the dielectric properties of the composite.^41,55^

When applying effective medium theory to the PDA-GO/LM/PDMS composite, we considered two conditions. Figure S6a shows the results for the first condition in which PDMS functions as the matrix and PDA-GO and LM co-filler are added to the PDMS composites. Nan’s fit is the only prediction that fit well with the experimental data using this approach. Meanwhile, Figure S6b presents the results that consider PDA-GO/PDMS as the matrix and LM as the filler added to the PDA-GO/PDMS composites. All of the EMT models give lower values of the dielectric constant of composites especially at high LM concentrations. This suggests that the interface between LM and PDA-GO, the homogeneity of the distribution of the filler, and the size of the filler particles all have a potentially significant influence on the dielectric properties of the composites.^56^ The significant differences in morphology and density between PDA-GO and LM also make it difficult to include all of the factors in the EMT models considered here.

**Figure 4:**
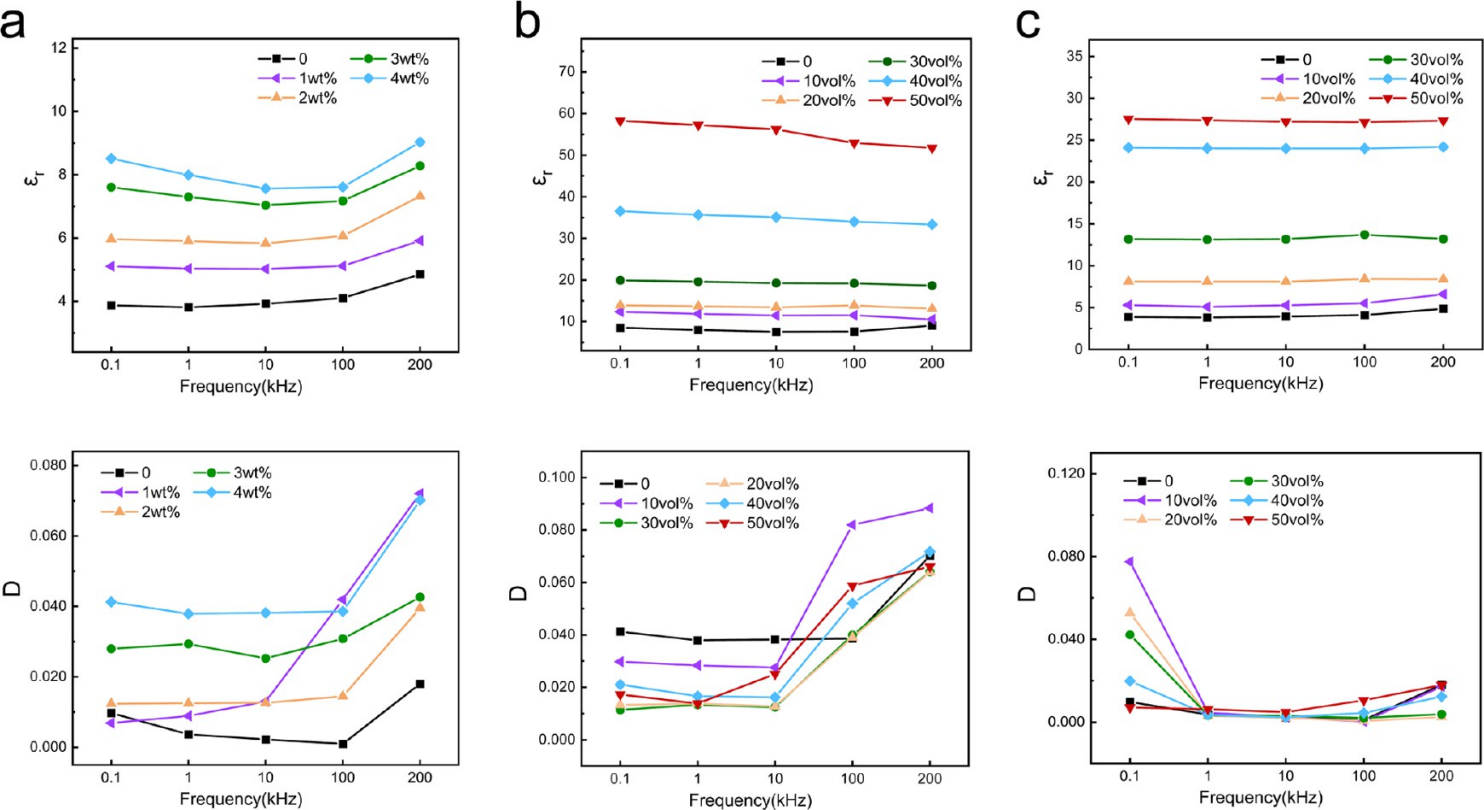
Frequency dependence of the dielectric constant and dissipation factor at different filler contents: (a) PDA-GO/PDMS composite with different PDA-GO contents, (b) PDA-GO/LM/PDMS composite with different LM contents, and (c) LM/PDMS composite with different LM contents.

To understand the frequency-dependent dielectric properties of the soft composites, we test the ε_r_ and *D* of the composites across the frequency range of 0.1–200 kHz. Figure 4a presents a plot of the effective dielectric constant as a function of testing frequency for PDA-GO contents from 0 to 4 wt %, showing that all of the PDA-GO/PDMS composites show a similar tendency of the dielectric properties across the frequency range of 0.1–200 kHz. The dielectric constant exhibits a modest reduction as the frequency increases and with a subtle increment observed at 200 kHz. This behavior is particularly pronounced in samples with the highest PDA-GO concentration. Meanwhile, the dissipation factor also shows a similar dependency on the frequency. This phenomenon can be attributed to the dominant contribution of dipolar relaxation and the influence of PDA coating.^41,57^Figure 4c shows the dielectric constant and dissipation factor of LM/PDMS composites in the range of 0.1–200 kHz. All groups exhibit a uniform value of ε_r_ across the entire frequency range, which is consistent with a previous study.^34,36^ Adding EGaIn droplets to the fixed amount of PDA-GO/PDMS composite leads to an increase in permittivity, and the dissipation factor remains small (*D* < 0.1), which is shown in Figure 4b. The electrical dissipation factor, also known as the dielectric loss tangent, refers to the proportion of dielectric power lost to the reactive power of capacitance during alternating current (ac) oscillation.^36^ The small value of *D* means that adding LM will not have a significant influence on the stability of the PDA-GO/LM/PDMS composite. Figure 4b also shows that for the PDA-GO/LM/PDMS composite with a Φ_LM_ of 50 vol % and a Φ_PDA-GO_ of 4 wt %, the dielectric constant decreases at high frequencies. The reason for this is that Maxwell–Wagner–Sillars (MWS) polarization cannot contribute as significantly to the high-frequency dielectric permittivity as it does to the low-frequency dielectric permittivity. As the MWS polarization is associated with the charging and discharging processes in the interfacial region of the composites, the charging–discharging cycle cannot be fully completed at higher frequencies.^41,55^

### Mechanical Properties of Multimaterial Composites

**Figure 5:**
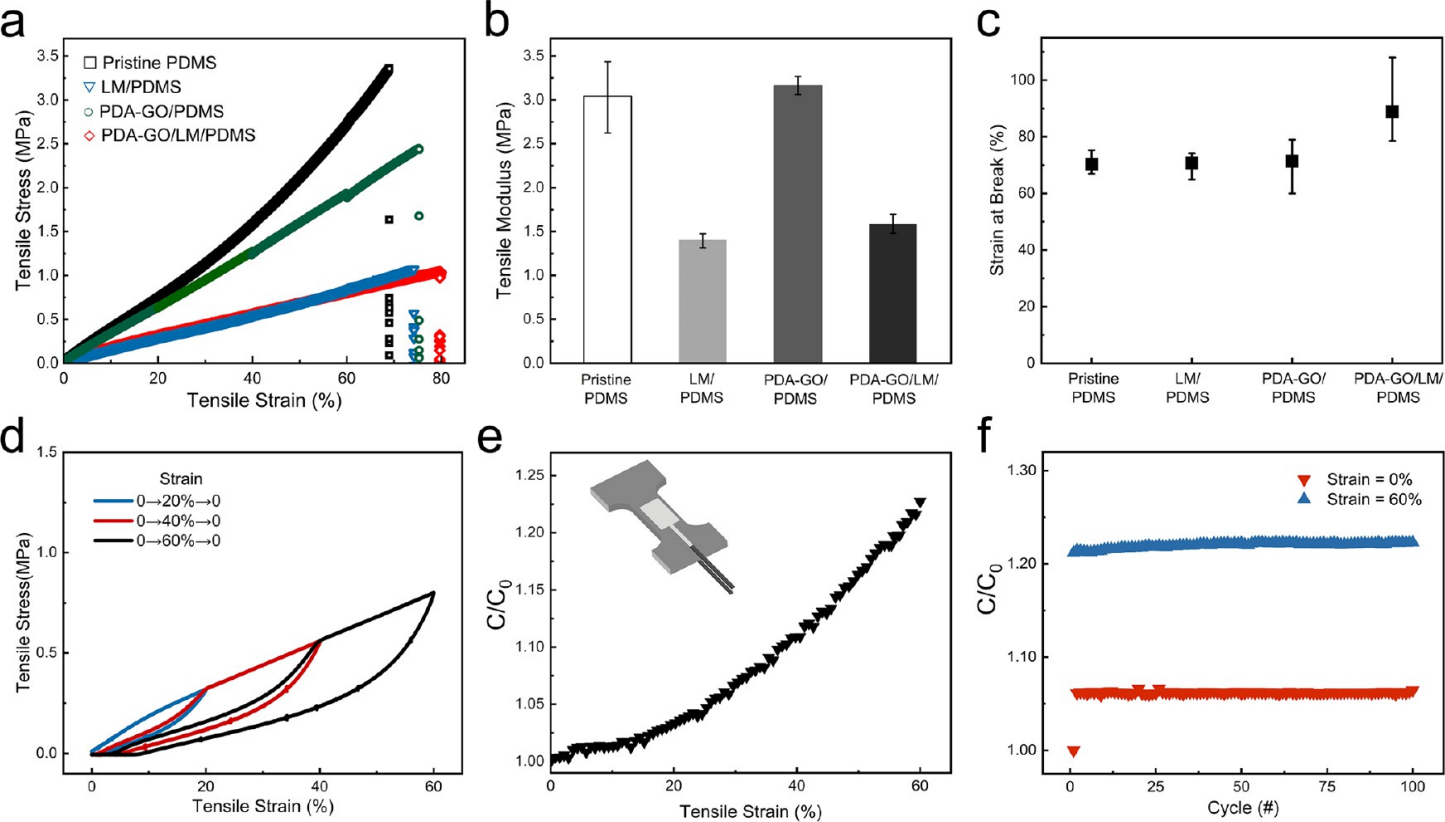
Mechanical and electromechanical characterization of PDMS composites with different fillers. (a) Plot of stress vs strain for the PDMS composites with different fillers tested until failure. (b) Plot of the tensile modulus (measured to 10% strain) for the different PDMS composites. (c) Strain at break for the different PDMS composites. (d) Stress–strain curves of the PDA-GO/LM/PDMS composite for cyclic tensile loading. (e) Plot of the ratio of capacitance (*C*) to initial capacitance (*C*_0_) vs strain for the PDA-GO/LM/PDMS composite. (f) Cyclic testing of the PDA-GO/LM/PDMS composite to 60% strain over 100 cycles.

The mechanical properties of the different classes of PDMS-based material systems are shown in Figure 5. These measurements show how PDA-GO and LM inclusions influence the stiffness and strain at break of the composites. Three specimens were tested for each class of materials. Figure 5a presents representative stress–strain curves for stretching to 100% strain. From these data, the influence of the stiffness of the rigid and liquid inclusions is studied by measuring the tensile modulus in the low-strain regime (0–10% strain) shown in Figure 5b. Adding 4 wt % PDA-GO to PDMS does not appear to alter the stiffness in a statistically significant manner. However, adding 50 vol % LM to PDMS significantly decreases its stiffness from 3.04 to 1.41 MPa. In contrast, adding PDA-GO results in a PDA-GO/LM/PDMS composite that has an only slightly greater modulus compared to that of LM/PDMS. Such a decrease in stiffness is consistent with Eshelby’s classical theory of inclusions. This theory suggests that a composite with liquid inclusions should have an elastic modulus of *E*_c_ = *E*_m_/(1 + 5ϕ/3),^58^ where *E*_c_ and *E*_m_ are the elastic modulus of the composite and matrix, respectively, and the mechanical resistance of the LM inclusions is ignored. Previously, Pan et al. mixed LM with PDMS and also observed a decrease in the tensile modulus when the diameter of LM inclusions was ∼10 μm,^36^ in accordance with Eshelby’s theory. Likewise, we find that the average LM inclusion size in LM/PDMS and PDA-GO/LM/PDMS composites to be 11.6 and 9.5 μm, respectively (Figure S3). With PDA-GO added, the LM droplets appear smaller, and this may due to PDA-GO/LM/PDMS having a higher viscosity that results in smaller LM droplets, which is consistent with previous studies.^59,60^

Figure 5c shows that the PDA-GO/LM/PDMS composites can stretch to strains on the order of ∼80%, similar to that of the unfilled elastomer. Following an initial loading cycle, the PDA-GO/LM/PDMS composites exhibit very low mechanical hysteresis when comparing subsequent loading and unloading curves (Figure 5d). The difference in the stress–strain curves for the initial loading cycle is attributed to Mullin’s effect,^61^ and we observe negligible internal friction or losses due to inelastic deformation in subsequent loading cycles.

Meanwhile, when 4 wt % PDA-GO is dispersed in LM/PDMS composites, the density of the composites decreases from ∼3.61 to ∼3.45. In other words, adding PDA-GO to LM/PDMS composites increases the electrical permittivity without increasing its density. In contrast, adding additional liquid metal to further enhance permittivity can result in substantial increases in density due to the relatively high specific gravity of EGaIn compared to that of PDMS.

### Electrical Response to Mechanical Deformation and Stability

The electrical response of the dielectric material to mechanical deformation is an important parameter for applications as a soft transducer. A parallel-plate capacitor composed of a PDA-GO/LM/PDMS dielectric and EGaIn electrodes encapsulated in a silicone elastomer (Figure 5e, inset) is used to measure electromechanical coupling during stretching. Figure 5e presents the results of normalized capacitance *C*/*C*_0_ as a function of tensile strain, where *C*_0_ is the capacitance at 0% strain. It is shown that the capacitance increases almost linearly with tensile strain. Tutika et al. measured the electrical response of multilayer LM/PDMS composites, which showed the change in sensor signal (*C*/*C*_0_) per change in strain (ε) is 0.8 for larger (*d* = 80 and 20 μm) LM droplets and 1.5 for smaller (*d* = 1 μm) LM droplets.^37^Figure 5f shows the cyclic testing results of *C*/*C*_0_ for 60% strain loading over 100 cycles to indicate the durability of the PDA-GO/LM/PDMS composite. The results show that the capacitance increases slightly at both 0% and 60% strain during the first cycle while remaining constant during the remaining 100 cycles and suggest that the PDA-GO/LM/PDMS composite is a stable and stretchable dielectric material.

## Conclusion

In this work, we introduce a PDA-GO/LM/PDMS elastomer composite and examine its dielectric and mechanical properties. An enhancement of 1400% in electrical permittivity is demonstrated by adding 4 wt % PDA-GO and 50 vol % LM to a soft PDMS matrix. The effective dielectric constant of this multiphase composite approximately follows the same trends predicted by effective medium theory. In addition, we examine the morphology and mechanical properties that arise from the incorporation of rigid and liquid phase inclusions. Lastly, we measure electromechanical coupling between elastic deformation and capacitance and show how the dielectric elastomer can function as a strain-sensing transducer. The combination of enhanced dielectric permittivity and rubber-like mechanical properties makes this composite a promising alternative to existing dielectric elastomer architectures that use liquid metal inclusions.

## Experimental Section

### Materials

Flake graphite, potassium permanganate (KMnO_4_), sodium nitrate (NaNO_3_), sulfuric acid (H_2_SO_4_), a hydrogen peroxide solution (H_2_O_2_), hydrochloric acid (HCl), dopamine hydrochloride, and tris(hydroxymethyl)aminomethane (Tris) were all purchased from Sigma-Aldrich. PDMS (Sylgard 184) was purchased from Dow Corning. EGaIn (75 wt % Ga and 25 wt % In) was prepared by mixing raw Ga and In (Rotometals Inc.) at 195 °C and stirring for 12 h.

### Graphene Oxide Functionalized by DA Self-Polymerization

Hummer’s method was used to synthesize graphene oxide from graphite. Briefly, graphite (2 g) and NaNO_3_ (1 g) were mixed with 46 mL of H_2_SO_4_ in an ice bath, followed by the slow addition of 6.0 g of KMnO_4_. After the addition of KMnO_4_, the solution in a beaker was heated to 35 °C in a water bath and stirred vigorously for 60 min. Deionized (DI) water (90 mL) was added to the suspension, and the temperature was increased to 95 °C. The bath was kept at this temperature for 60 min while the sample was stirred. The reaction ended upon addition of DI water (200 mL) and hydrogen peroxide (30%, 2 mL). The mixture was then centrifuged at 3000 rpm for 10 min to remove the residual graphite and washed with diluted HCl (1 mol/L) solution and deionized water repeatedly. After the mixture turned into two phases, the upper phase was removed and dried in an oven overnight at 60 °C.^62^

GO (100 mg) and DI water (100 mL) were added to a bottle and then ultrasonicated at 40% amplitude for 1 h. Then the GO solution and 100 mg of DA hydrochloride were added to 200 mL of a 10 mM Tris-HCl solution, followed by vigorous stirring at 60 °C for 24 h. After the reaction had reached completion, the dispersion was centrifuged at 12000 rpm for 20 min, washed thrice with DI water, and dried by lyophilization.^41^

### Preparation of Composites by Shear Mixing

All composites were formed using PDMS (Sylgard 184, Dow Corning) with a base:curing agent ratio of 10:1. Composites were made with 0, 10, 20, 30, 40, and 50 vol % liquid metal (EGaIn) and 0, 1, 2, 3, and 4 wt % PDA-GO. All composites were shear mixed for 6 min in a planetary centrifugal mixer (AR-100, THINKY Crop.) The uncured polymer was then deposited onto a substrate using a thin film applicator (ZUA 2000.150, Zehntner). After deposition, the material was cured for 12 h at 90 °C.

### Characterizations

The FTIR spectrum (PerkinElmer) was measured in the range of 500–4000 cm^–1^. Scanning electron microscopy (SEM) images of PDA-GO and the cross section of the PDA-GO/LM/PDMS composite were obtained using a Quanta 600 FEG instrument equipped with an energy-dispersive X-ray spectroscope and an accelerating voltage of 20 kV.

*Measurement of Dielectric Properties*. A 2 cm × 2 cm thin film (∼300 μm) of the composites was created with a thin film applicator on a metal plate. The 1 cm × 1 cm EGaIn contact pads were then applied to the surface of the composites to act as the top plate of the capacitor. Then, the benchtop LCR meter (889B, BK Precision) was used to measure the capacitance and dissipation factor, and the corresponding effective dielectric constant was calculated. At least three samples of each specimen were prepared, and five measurements were taken for each sample.

*Measurement of Mechanical Properties*. A thin film (∼500 μm) of composites with dogbone geometry (ASTM D638_ Type V) was created on a metal plate using a thin film applicator. To prevent adhesion, the metal plate was coated with mold release (Smooth-On Universal Mold Release) prior to deposition. The same process was used to cure the composite film. Next, samples were tested on a materials testing system (Instron 5969, Illinois Tool Works Inc.) with a 50 N load cell and an extension rate of 1 mm s^–1^.

*Measurement of Electromechanical Coupling*. Electromechanical coupling samples with dogbone geometry and a EGaIn electrode were fabricated in layers and stretched on an Instron 5969 mechanical testing machine. Two conductive fiber strips were used as the connector to the benchtop LCR meter. The capacitance data were collected with the benchtop LCR meter (889B, BK Precision).

## Supporting Information Available

Sample preparation for breakdown strength measurement, FTIR spectra of pristine GO and PDA-GO, SEM image and EDS elemental mapping of PDA-GO, particle analysis of the LM/PDMS composite and PDA-GO/LM/PDMS composite, breakdown strength of LM/PDMS and PDA-GO/LM/PDMS composites, and EMT models for LM/PDMS, PDA-GO/PDMS and PDA-GO/LM/PDMS composites (PDF)

## References

[ref1] Nesser H.; Lubineau G. Strain sensing by electrical capacitive variation: From stretchable materials to electronic interfaces. Advanced Electronic Materials 2021, 7, 2100190 10.1002/aelm.202100190.

[ref2] Qin J.; Yin L.-J.; Hao Y.-N.; Zhong S.-L.; Zhang D.-L.; Bi K.; Zhang Y.-X.; Zhao Y.; Dang Z.-M. Flexible and stretchable capacitive sensors with different microstructures. Adv. Mater. 2021, 33, 2008267 10.1002/adma.202008267. 34240474

[ref3] Lazarus N.; Meyer C.; Bedair S.; Nochetto H.; Kierzewski I. Multilayer liquid metal stretchable inductors. Smart materials and structures 2014, 23, 085036 10.1088/0964-1726/23/8/085036.

[ref4] Yang Y.; Wu Y.; Li C.; Yang X.; Chen W. Flexible actuators for soft robotics. Advanced Intelligent Systems 2020, 2, 1900077 10.1002/aisy.201900077.

[ref5] Majidi C.; Alizadeh K.; Ohm Y.; Silva A.; Tavakoli M. Liquid metal polymer composites: From printed stretchable circuits to soft actuators. Flexible and Printed Electronics 2022, 7, 013002 10.1088/2058-8585/ac515a.

[ref6] Gupta U.; Qin L.; Wang Y.; Godaba H.; Zhu J. Soft robots based on dielectric elastomer actuators: A review. Smart Materials and Structures 2019, 28, 103002 10.1088/1361-665X/ab3a77.

[ref7] Ahn J.; Gu J.; Choi J.; Han C.; Jeong Y.; Park J.; Cho S.; Oh Y. S.; Jeong J.-H.; Amjadi M.; et al. A Review of Recent Advances in Electrically Driven Polymer-Based Flexible Actuators: Smart Materials, Structures, and Their Applications. Advanced Materials Technologies 2022, 7, 2200041 10.1002/admt.202200041.

[ref8] Erdem Ö.; Derin E.; Zeibi Shirejini S.; Sagdic K.; Yilmaz E. G.; Yildiz S.; Akceoglu G. A.; Inci F. Carbon-Based Nanomaterials and Sensing Tools for Wearable Health Monitoring Devices. Advanced Materials Technologies 2022, 7, 2100572 10.1002/admt.202100572.

[ref9] Surmenev R. A.; Chernozem R. V.; Pariy I. O.; Surmeneva M. A. A review on piezo-and pyroelectric responses of flexible nano-and micropatterned polymer surfaces for biomedical sensing and energy harvesting applications. Nano Energy 2021, 79, 105442 10.1016/j.nanoen.2020.105442.

[ref10] Zadan M.; Chiew C.; Majidi C.; Malakooti M. H. Liquid metal architectures for soft and wearable energy harvesting devices. Multifunctional Materials 2021, 4, 012001 10.1088/2399-7532/abd4f0.

[ref11] Wang C.; Xia K.; Wang H.; Liang X.; Yin Z.; Zhang Y. Advanced carbon for flexible and wearable electronics. Advanced materials 2019, 31, 1801072 10.1002/adma.201801072. 30300444

[ref12] Zu W.; Ohm Y.; Carneiro M. R.; Vinciguerra M.; Tavakoli M.; Majidi C. A Comparative Study of Silver Microflakes in Digitally Printable Liquid Metal Embedded Elastomer Inks for Stretchable Electronics. Advanced Materials Technologies 2022, 7, 2200534 10.1002/admt.202200534.

[ref13] Sim K.; Rao Z.; Ershad F.; Yu C. Rubbery electronics fully made of stretchable elastomeric electronic materials. Adv. Mater. 2020, 32, 1902417 10.1002/adma.201902417. 31206819

[ref14] Wang J.; Gao D.; Lee P. S. Recent progress in artificial muscles for interactive soft robotics. Adv. Mater. 2021, 33, 2003088 10.1002/adma.202003088. 33108022

[ref15] Wang X.; Guo R.; Liu J. Liquid metal based soft robotics: materials, designs, and applications. Adv. Mater. Technol. 2019, 4, 1970009 10.1002/admt.201970009.

[ref16] Majidi C. Soft-matter engineering for soft robotics. Advanced Materials Technologies 2018, 4, 1800477 10.1002/admt.201800477.

[ref17] Moretti G.; Rosset S.; Vertechy R.; Anderson I.; Fontana M. A review of dielectric elastomer generator systems. Advanced Intelligent Systems 2020, 2, 2000125 10.1002/aisy.202000125.

[ref18] Coyle S.; Majidi C.; LeDuc P.; Hsia K. J. Bio-inspired soft robotics: Material selection, actuation, and design. Extreme Mechanics Letters 2018, 22, 51–59. 10.1016/j.eml.2018.05.003.

[ref19] Park S.; Mondal K.; Treadway R. M. III; Kumar V.; Ma S.; Holbery J. D.; Dickey M. D. Silicones for stretchable and durable soft devices: Beyond Sylgard-184. ACS Appl. Mater. Interfaces 2018, 10, 11261–11268. 10.1021/acsami.7b18394. 29578686

[ref20] Park S.; Thangavel G.; Parida K.; Li S.; Lee P. S. A stretchable and self-healing energy storage device based on mechanically and electrically restorative liquid-metal particles and carboxylated polyurethane composites. Adv. Mater. 2019, 31, 1805536 10.1002/adma.201805536. 30387213

[ref21] Li G.; Chen X.; Zhou F.; Liang Y.; Xiao Y.; Cao X.; Zhang Z.; Zhang M.; Wu B.; Yin S.; et al. Self-powered soft robot in the Mariana Trench. Nature 2021, 591, 66–71. 10.1038/s41586-020-03153-z. 33658693

[ref22] Lai Y.-C.; Lu H.-W.; Wu H.-M.; Zhang D.; Yang J.; Ma J.; Shamsi M.; Vallem V.; Dickey M. D. Elastic multifunctional liquid–metal fibers for harvesting mechanical and electromagnetic energy and as self-powered sensors. Adv. Energy Mater. 2021, 11, 2100411 10.1002/aenm.202100411.

[ref23] Tutika R.; Haque A.; Bartlett M. D. Self-healing liquid metal composite for reconfigurable and recyclable soft electronics. Commun. Mater. 2021, 2, 64 10.1038/s43246-021-00169-4.

[ref24] Goyal R.; Katkade S.; Mule D. Dielectric, mechanical and thermal properties of polymer/BaTiO3 composites for embedded capacitor. Composites Part B: Engineering 2013, 44, 128–132. 10.1016/j.compositesb.2012.06.019.

[ref25] Liu L.; Zhang W.; Ning N.; Zhang L. A self-healing dielectric supramolecular elastomer modified by TiO2/urea particles. Chemical Engineering Journal 2019, 375, 121993 10.1016/j.cej.2019.121993.

[ref26] Li H.; Liu G.; Liu B.; Chen W.; Chen S. Dielectric properties of polyimide/Al2O3 hybrids synthesized by in-situ polymerization. Mater. Lett. 2007, 61, 1507–1511. 10.1016/j.matlet.2006.07.063.

[ref27] Lu J.; Moon K.-S.; Xu J.; Wong C. Synthesis and dielectric properties of novel high-K polymer composites containing in-situ formed silver nanoparticles for embedded capacitor applications. J. Mater. Chem. 2006, 16, 1543–1548. 10.1039/b514182f.

[ref28] Chen Z.; Xie L.; Huang X.; Li S.; Jiang P. Achieving large dielectric property improvement in polymer/carbon nanotube composites by engineering the nanotube surface via atom transfer radical polymerization. Carbon 2015, 95, 895–903. 10.1016/j.carbon.2015.09.020.

[ref29] Panahi-Sarmad M.; Chehrazi E.; Noroozi M.; Raef M.; Razzaghi-Kashani M.; Haghighat Baian M. A. Tuning the surface chemistry of graphene oxide for enhanced dielectric and actuated performance of silicone rubber composites. ACS Applied Electronic Materials 2019, 1, 198–209. 10.1021/acsaelm.8b00042.

[ref30] Wang M.; Duan X.; Xu Y.; Duan X. Functional three-dimensional graphene/polymer composites. ACS Nano 2016, 10, 7231–7247. 10.1021/acsnano.6b03349. 27403991

[ref31] Song S.; Zhai Y.; Zhang Y. Bioinspired graphene oxide/polymer nanocomposite paper with high strength, toughness, and dielectric constant. ACS Appl. Mater. Interfaces 2016, 8, 31264–31272. 10.1021/acsami.6b08606. 27782385

[ref32] Li Y.; Cui Y.; Zhang M.; Li X.; Li R.; Si W.; Sun Q.; Yu L.; Huang C. Ultrasensitive Pressure Sensor Sponge Using Liquid Metal Modulated Nitrogen-Doped Graphene Nanosheets. Nano Lett. 2022, 22, 2817–2825. 10.1021/acs.nanolett.1c04976. 35333055

[ref33] Yang Z.; Pang Y.; Han X.-l.; Yang Y.; Ling J.; Jian M.; Zhang Y.; Yang Y.; Ren T.-L. Graphene textile strain sensor with negative resistance variation for human motion detection. ACS Nano 2018, 12, 9134–9141. 10.1021/acsnano.8b03391. 30134097

[ref34] Bartlett M. D.; Fassler A.; Kazem N.; Markvicka E. J.; Mandal P.; Majidi C. Stretchable, high-k dielectric elastomers through liquid-metal inclusions. Adv. Mater. 2016, 28, 3726–3731. 10.1002/adma.201506243. 27007888

[ref35] Bartlett M. D.; Kazem N.; Powell-Palm M. J.; Huang X.; Sun W.; Malen J. A.; Majidi C. High thermal conductivity in soft elastomers with elongated liquid metal inclusions. Proc. Natl. Acad. Sci. U. S. A. 2017, 114, 2143–2148. 10.1073/pnas.1616377114. 28193902 PMC5338550

[ref36] Pan C.; Markvicka E. J.; Malakooti M. H.; Yan J.; Hu L.; Matyjaszewski K.; Majidi C. A liquid-metal–elastomer nanocomposite for stretchable dielectric materials. Adv. Mater. 2019, 31, 1900663 10.1002/adma.201900663. 30997710

[ref37] Tutika R.; Kmiec S.; Haque A. T.; Martin S. W.; Bartlett M. D. Liquid metal–elastomer soft composites with independently controllable and highly tunable droplet size and volume loading. ACS Appl. Mater. Interfaces 2019, 11, 17873–17883. 10.1021/acsami.9b04569. 31007016

[ref38] Malakooti M. H.; Bockstaller M. R.; Matyjaszewski K.; Majidi C. Liquid metal nanocomposites. Nanoscale Advances 2020, 2, 2668–2677. 10.1039/D0NA00148A. 36132412 PMC9419082

[ref39] Dang Z.-M.; Yuan J.-K.; Zha J.-W.; Zhou T.; Li S.-T.; Hu G.-H. Fundamentals, processes and applications of high-permittivity polymer–matrix composites. Prog. Mater. Sci. 2012, 57, 660–723. 10.1016/j.pmatsci.2011.08.001.

[ref40] Musto P.; Russo P.; Cimino F.; Acierno D.; Lupò G.; Petrarca C. Dielectric behavior of biopolymer based composites containing multi wall carbon nanotubes: Effect of filler content and aspect ratio. Eur. Polym. J. 2015, 64, 170–178. 10.1016/j.eurpolymj.2015.01.010.

[ref41] Ning N.; Ma Q.; Liu S.; Tian M.; Zhang L.; Nishi T. Tailoring dielectric and actuated properties of elastomer composites by bioinspired poly (dopamine) encapsulated graphene oxide. ACS Appl. Mater. Interfaces 2015, 7, 10755–10762. 10.1021/acsami.5b00808. 25938262

[ref42] Song Y.; Shen Y.; Liu H.; Lin Y.; Li M.; Nan C.-W. Improving the dielectric constants and breakdown strength of polymer composites: Effects of the shape of the BaTiO 3 nanoinclusions, surface modification and polymer matrix. J. Mater. Chem. 2012, 22, 16491–16498. 10.1039/c2jm32579a.

[ref43] He X.; Zhou J.; Jin L.; Long X.; Wu H.; Xu L.; Gong Y.; Zhou W. Improved dielectric properties of thermoplastic polyurethane elastomer filled with core–shell structured PDA@ TiC particles. Materials 2020, 13, 3341 10.3390/ma13153341. 32727113 PMC7435405

[ref44] Yang D.; Kong X.; Ni Y.; Xu Y.; Huang S.; Shang G.; Xue H.; Guo W.; Zhang L. Enhancement of dielectric performance of polymer composites via constructing BaTiO3–Poly (dopamine)–Ag nanoparticles through mussel-inspired surface functionalization. ACS omega 2018, 3, 14087–14096. 10.1021/acsomega.8b02367. 31458101 PMC6644546

[ref45] Fu L.; Lai G.; Jia B.; Yu A. Preparation and electrocatalytic properties of polydopamine functionalized reduced graphene oxide-silver nanocomposites. Electrocatalysis 2015, 6, 72–76. 10.1007/s12678-014-0219-9.

[ref46] Palanisamy S.; Thirumalraj B.; Chen S.-M.; Wang Y.-T.; Velusamy V.; Ramaraj S. K. A facile electrochemical preparation of reduced graphene oxide@ polydopamine composite: a novel electrochemical sensing platform for amperometric detection of chlorpromazine. Sci. Rep. 2016, 6, 33599 10.1038/srep33599. 27650697 PMC5030524

[ref47] Zhao Z.; Guo L.; Feng L.; Lu H.; Xu Y.; Wang J.; Xiang B.; Zou X. Polydopamine functionalized graphene oxide nanocomposites reinforced the corrosion protection and adhesion properties of waterborne polyurethane coatings. Eur. Polym. J. 2019, 120, 109249 10.1016/j.eurpolymj.2019.109249.

[ref48] Cui M.; Ren S.; Zhao H.; Xue Q.; Wang L. Polydopamine coated graphene oxide for anticorrosive reinforcement of water-borne epoxy coating. Chemical Engineering Journal 2018, 335, 255–266. 10.1016/j.cej.2017.10.172.

[ref49] Xia X.; Wang Y.; Zhong Z.; Weng G. J. A frequency-dependent theory of electrical conductivity and dielectric permittivity for graphene-polymer nanocomposites. Carbon 2017, 111, 221–230. 10.1016/j.carbon.2016.09.078. 28337974

[ref50] Luo H.; Wu Z.; Zhou X.; Yan Z.; Zhou K.; Zhang D. Enhanced performance of P (VDF-HFP) composites using two-dimensional BaTiO3 platelets and graphene hybrids. Compos. Sci. Technol. 2018, 160, 237–244. 10.1016/j.compscitech.2018.03.034.

[ref51] Nan C. W.; Birringer R.; Clarke D. R.; Gleiter H. J. Appl. Phys. 1997, 81, 6692 10.1063/1.365209.

[ref52] Bruggeman V. D. Berechnung verschiedener physikalischer Konstanten von heterogenen Substanzen. I. Dielektrizitätskonstanten und Leitfähigkeiten der Mischkörper aus isotropen Substanzen. Annalen der physik 1935, 416, 636–664. 10.1002/andp.19354160705.

[ref53] Choy T. C. Effective medium theory: principles and applications; Oxford University Press, 2015; Vol. 165.

[ref54] Aspnes D. Local-field effects and effective-medium theory: a microscopic perspective. American Journal of Physics 1982, 50, 704–709. 10.1119/1.12734.

[ref55] Almadhoun M. N.; Hedhili M. N.; Odeh I. N.; Xavier P.; Bhansali U. S.; Alshareef H. N. Influence of stacking morphology and edge nitrogen doping on the dielectric performance of graphene–polymer nanocomposites. Chem. Mater. 2014, 26, 2856–2861. 10.1021/cm5004565.

[ref56] Luo B.; Wang X.; Zhao Q.; Li L. Synthesis, characterization and dielectric properties of surface functionalized ferroelectric ceramic/epoxy resin composites with high dielectric permittivity. Compos. Sci. Technol. 2015, 112, 1–7. 10.1016/j.compscitech.2015.02.018.

[ref57] Yang D.; Kong X.; Ni Y.; Ruan M.; Huang S.; Shao P.; Guo W.; Zhang L. Improved mechanical and electrochemical properties of XNBR dielectric elastomer actuator by poly (dopamine) functionalized graphene nano-sheets. Polymers 2019, 11, 218 10.3390/polym11020218. 30960201 PMC6419049

[ref58] Eshelby J. D. The determination of the elastic field of an ellipsoidal inclusion, and related problems. Proc. R. Soc. A 1957, 241, 376–396. 10.1098/rspa.1957.0133.

[ref59] Koh A.; Sietins J.; Slipher G.; Mrozek R. Deformable liquid metal polymer composites with tunable electronic and mechanical properties. J. Mater. Res. 2018, 33, 2443–2453. 10.1557/jmr.2018.209.

[ref60] Bury E.; Koh A. S. Multimodal Deformation of Liquid Metal Multimaterial Composites as Stretchable, Dielectric Materials for Capacitive Pressure Sensing. ACS Appl. Mater. Interfaces 2022, 14, 13678–13691. 10.1021/acsami.1c21734. 35258947

[ref61] Mullins L. Softening of rubber by deformation. Rubber chemistry and technology 1969, 42, 339–362. 10.5254/1.3539210.

[ref62] Guerrero-Contreras J.; Caballero-Briones F. Graphene oxide powders with different oxidation degree, prepared by synthesis variations of the Hummers method. Mater. Chem. Phys. 2015, 153, 209–220. 10.1016/j.matchemphys.2015.01.005.

