# Supplementary material for: Dielectric Elastomers with Liquid Metal and Polydopamine-Coated Graphene Oxide Inclusions
Source: ACS Appl Mater Interfaces. 2023 May 15;15(20):24769–76. doi: 10.1021/acsami.2c21994 (PMC10214383; doi:10.1021/acsami.2c21994)
Supplement: Supplementary file 1 — am2c21994_si_001.pdf [file am2c21994_si_001.pdf]

Support information: Dielectric elastomers with liquid metal and polydopamine-coated graphene oxide inclusions

Yafeng Hu† and Carmel Majidi*,†,‡

†*Material Science & Engineering, Carnegie Mellon University, Pittsburgh, PA USA*

‡*Mechanical Engineering, Carnegie Mellon University, Pittsburgh, PA USA*

E-mail: cmajidi@andrew.cmu.edu

Sample preparation for breakdown strength measurement:

A 7cm × 7cm thin film (∼300 μm) sample of PDA-GO/LM/PDMS composite was created from uncured composites using a thin film applicator (ZUA 2000.150; Zehntner) on a stainless steel sheet (mirror-like surface finish; McMaster-Carr). The steel sheet was placed in a 90°C oven for 12 hours to cure the composite. Next, circular EGaIn electrodes with diameter of 5 mm were applied on the film surface as the top plate of capacitor. A metal wire probe was merged into the EGaIn electrode and the other lead was connected to the steel sheet for the connection between the sample and a high voltage power supply (PS 375, Stanford Research Systems) to supply DC voltage. At least 16 data points per sample were collected.

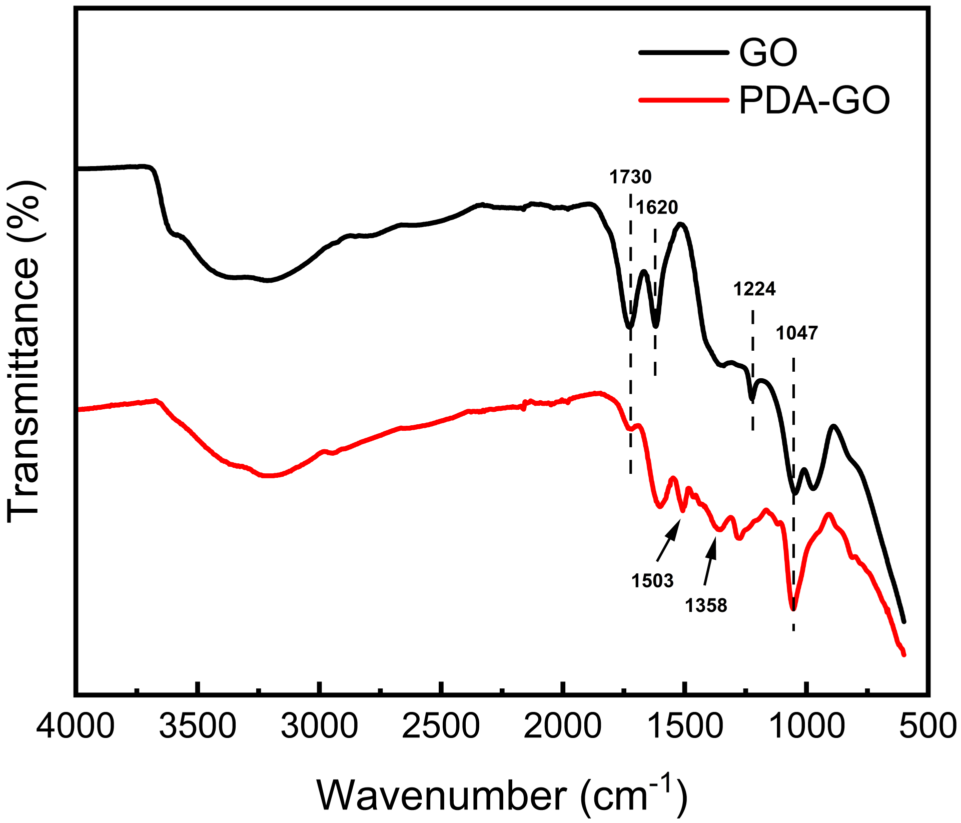

Figure S1. FTIR spectra of the pristine GO and PDA-GO.

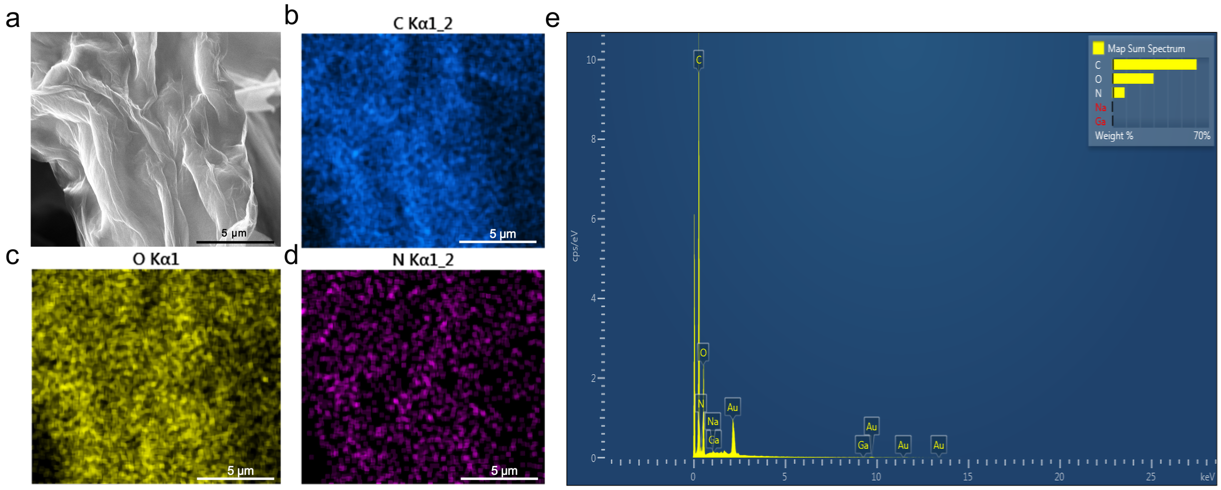

Figure S2. SEM image of (a) PDA-GO, corresponding EDS elemental mapping of (b) carbon, (c) oxygen and (d) nitrogen. (e) EDS spectrum of PDA-GO.

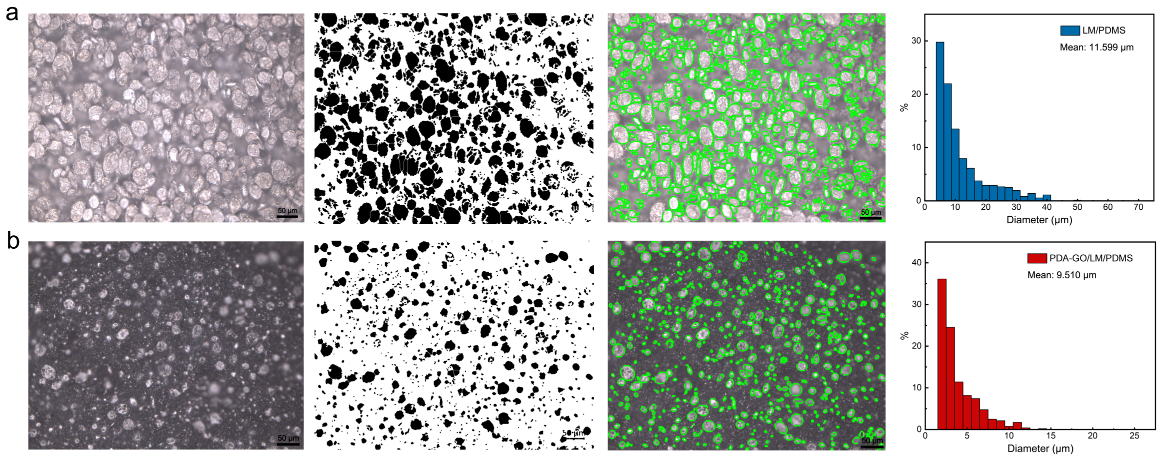

Figure S3. Particle analysis of (a) LM/PDMS composite and (b) PDA-GO/LM/PDMS composite. Column 1, Optical micrographs. The scale bar is 50 μm. Column 2, Thresholded image. Column 3, Ellipses fit to the particles in the thresholded image overlaid on the optical micrographs. Column 4, histogram of the percent of analyzed droplets versus major radii.

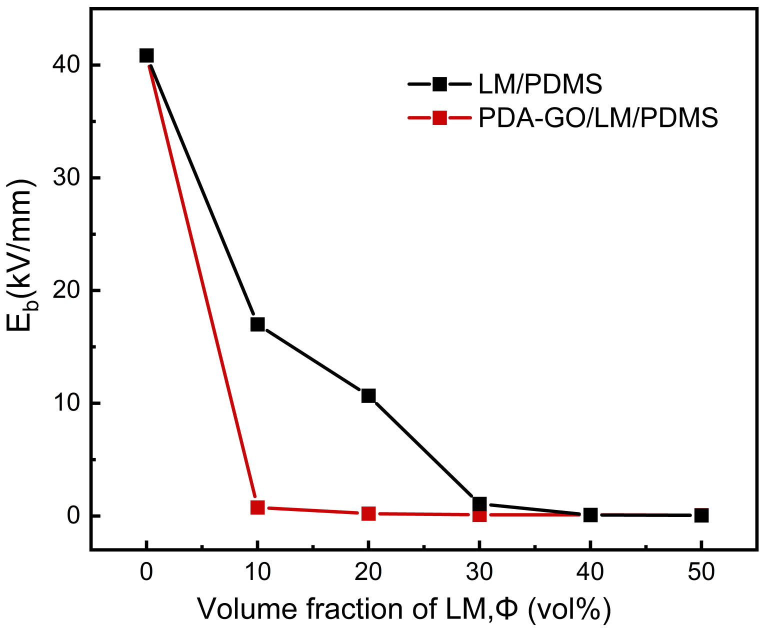

Figure S4. Breakdown strength of LM/PDMS and PDA-GO/LM/PDMS composites as a function of LM filler content.

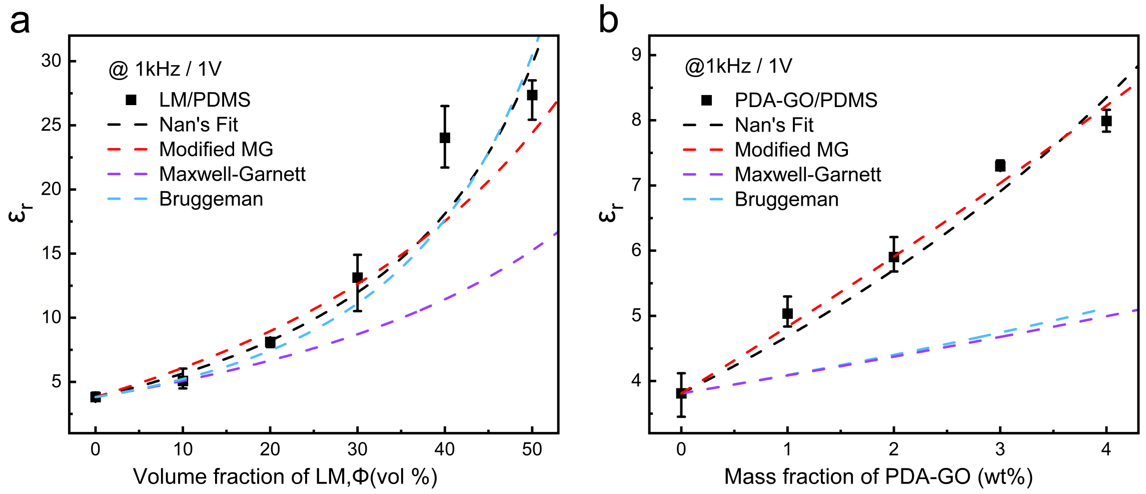

Figure S5. Different EMT Models for (a) LM/PDMS and (b) PDA-GO/PDMS composites. Symbols with error bars are the experimental data. Black dashed curve is Nan et al[1,2] formulation (α value for each group: α_a=0.099, α_b=0.288) . Red dashed line curve is Modified Maxwell-Garnett (Modified MG)[3] effective medium theory (The shape factor of the filler n for each group: n_a=0.185, n_b=0.089). Purple dashed curve is Maxwell-Garnett[4] effective medium theory. Blue dashed curve is Bruggemann effective medium theory.[5]

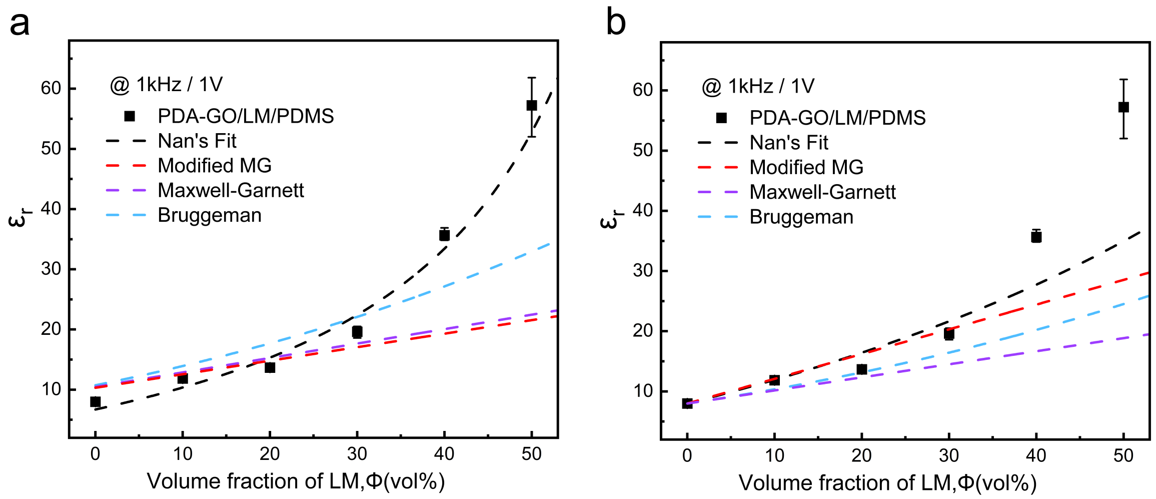

Figure S6. Different EMT Models for PDA-GO/LM/PDMS composites. (a) Considering as PDA-GO and LM co-filler added into PDMS matrix. (b) Considering as LM single filler added into PDA-GO/PDMS matrix. Symbols with error bars are the experimental data. Black dashed curve is Nan et al[1,2] formulation (α value for each group: α_a=0.217, α_b=0.159) . Red dashed line curve is Modified Maxwell-Garnett (Modified MG)[3] effective medium theory (The shape factor of the filler n for each group: n_a=0.355, n_b=0.176). Purple dashed curve is Maxwell-Garnett[4] effective medium theory. Blue dashed curve is Bruggemann effective medium theory.[5]

References

(1) Nan, C. W., Birringer R., Clarke DR, Gleiter H. *J. Appl. Phys* **1997**, *81*, 6692.

(2) Pan, C.; Markvicka, E. J.; Malakooti, M. H.; Yan, J.; Hu, L.; Matyjaszewski, K.; Majidi, C. A liquid-metal–elastomer nanocomposite for stretchable dielectric materials. *Advanced Materials* **2019**, *31*, 1900663.

(3) Tinga, W. R.; Voss, W.; Blossey, D. Generalized approach to multiphase dielectric mixture theory. *Journal of applied physics* **1973**, *44*, 3897–3902.

(4) Luo, B.; Wang, X.; Zhao, Q.; Li, L. Synthesis, characterization and dielectric properties

of surface functionalized ferroelectric ceramic/epoxy resin composites with high dielectric permittivity. *Composites Science and Technology* **2015**, *112*, 1–7.

(5) Aspnes, D. Local-field effects and effective-medium theory: a microscopic perspective. *American Journal of Physics* **1982**, *50*, 704–709.
